# Comparative efficacy and cognitive function of magnetic seizure therapy and electroconvulsive therapy for major depressive disorder: a systematic review and meta-analysis of randomized controlled trials

**DOI:** 10.3389/fpsyt.2026.1886955

**Published:** 2026-07-22

**Authors:** Yu-hang Chen, Xue Zhou, Jun-shan Chen

**Affiliations:** 1 Department of Operations Management, Chongqing Mental Health Center, Chongqing, China; 2 Pharmacy Department, Chongqing Mental Health Center, Chongqing, China

**Keywords:** electroconvulsive therapy, magnetic seizure therapy, major depressive disorder, meta-analysis, RCTs

## Abstract

**Background:**

Magnetic seizure therapy (MST), as a novel physical therapy technique, has demonstrated significant efficacy in the treatment of depression. Previous systematic reviews and meta-analyses have preliminarily confirmed its clinical value. This study conducts a meta-analysis based on updated randomized controlled trials to evaluate the differences in efficacy between MST and electroconvulsive therapy (ECT) in patients with major depressive disorder (MDD), as well as their effects on cognitive function.

**Methods:**

We conducted a systematic search of PubMed, Embase, the Cochrane Library, Web of Science, Ovid, ProQuest, and CNKI databases for randomized controlled trials published up to March 1, 2026. The selection of studies strictly followed the Cochrane Risk of Bias in Randomized Trials tool, and the meta-analysis was conducted in accordance with the Cochrane Systematic Reviewer’s Manual. Data from 429 patients across 6 studies were included in the quantitative synthesis.

**Results:**

The results showed that post-treatment depression scale scores in the MST group were 0.329 higher than those in the ECT group (95% CI: 0.119-0.539, I² = 9.4%, P = 0.0021), suggesting that the antidepressant efficacy of MST was inferior to that of ECT. In the neurocognitive subgroup analysis, MST performed significantly better than ECT in verbal fluency (SMD = 0.75, 95% CI: 0.19-1.31, P = 0.0088), immediate memory (SMD = 0.68, 95% CI: 0.35-1.01, P < 0.0001), delayed memory (SMD = 0.61, 95% CI: 0.28-0.94, P = 0.0003), and verbal learning (SMD = 0.57, 95% CI: 0.18-0.95, P = 0.0037).

**Conclusions:**

Our evidence-based study conducted a meta-analysis of the antidepressant efficacy of MST and its effects on cognitive function. The evidence from this study suggests that MST may be slightly less effective than ECT in treating depression but may offer advantages in terms of cognitive preservation; further research is needed to confirm these findings.

## Introduction

MDD is a severe mental disorder and the most common cause of disability worldwide, with a lifetime prevalence of nearly 20% (1). Epidemiological data show that between 2007 and 2017, the global prevalence of MDD rose significantly, with incidence increasing by approximately 13.0% over the decade (2). Furthermore, studies have reported that the recurrence rate within 10 years after a first depressive episode can be as high as 85% (3). MDD can severely impact patients’ daily work, lives, and interpersonal relationships, and may even lead to an increased risk of suicide (4).

MDD is a clinically heterogeneous syndrome, characterized by diverse symptoms and variable treatment responses. Overall, 30-50% of patients show an inadequate response to first-line treatments, which typically include antidepressants and cognitive behavioral therapy (5). Currently, ECT is widely recognized as one of the effective antidepressant treatments, particularly for MDD (6), with remission rates ranging from 40% to 70% (7). However, previous studies have found that ECT impairs patients’ cognitive function, particularly memory function (8).

MST is a treatment method that induces epileptic discharges and convulsive seizures through high-intensity, high-frequency repetitive transcranial magnetic stimulation (rTMS). It offers efficacy comparable to ECT but with greater safety (8). Compared to TMS, MST features higher magnetic field frequencies and more stable output voltages, making it more suitable for treating severe psychiatric disorders. Compared to ECT, MST can more accurately induce spatial currents on the surface of the cerebral cortex and selectively stimulate localized cortical areas without affecting deep brain nuclei (9).

Currently, conclusions regarding the efficacy and cognitive outcomes of MST for treating depression remain inconsistent. Early systematic reviews demonstrated that MST is effective in alleviating depressive symptoms in mood disorders and generally has fewer side effects than ECT (10). However, a subsequent systematic review noted that the evidence base regarding the efficacy of MST in treating treatment-resistant depression (TRD) remains limited and is insufficient to draw definitive conclusions; while the available evidence does not preclude the clinical use of MST in patients with TRD, this intervention should still be regarded as an experimental therapy in the exploratory phase (11). The small sample sizes in the two studies limit the generalizability of the results. In a recent systematic review, MST was again demonstrated to be an effective and well-tolerated treatment for depression (12); however, the fact that most studies were non-randomized studies reduces the certainty of the review’s conclusions. Cognitive impairment is a core symptom of MDD (13), and patients with depression exhibit moderate deficits in executive function, memory, and attention (14). The impact of MST on cognitive function remains controversial. A recent meta-analysis demonstrated for the first time that, compared to ECT, patients with depression treated with MST derive greater cognitive benefits (15). However, another systematic review indicated that findings regarding the adverse cognitive effects of MST are inconsistent (16). Therefore, a comprehensive evaluation of MST is necessary, based on evidence regarding its efficacy and effects on cognitive function.

In this latest systematic review and meta-analysis, we sought to evaluate the antidepressant efficacy of MST in patients with major depressive disorder and its impact on cognitive function. We synthesized data from relevant randomized controlled trials (RCTs) to provide valuable insights for further clinical research and the promotion of MST.

## Methods

### Study design

This systematic review and meta-analysis was conducted in accordance with the 2020 Preferred Reporting Items for Systematic Reviews and Meta-Analyses (PRISMA) guidelines (17). This manuscript has been registered in the PROSPERO database (CRD420251267533).

### Search strategy and data sources

We conducted a computerized search of PubMed, Embase, the Cochrane Library, Web of Science, Ovid, ProQuest, and CNKI databases for literature published from the inception of each database through March 1, 2026, as well as clinical trials with published results in ClinicalTrials. Keywords included depression, depressive disorder, major depressive disorder, MDD, magnetic seizure therapy, and MST. No language or year restrictions were applied (**Supplementary Material**).

### Studies selection

The two review authors (YHC, XZ) independently conducted an initial assessment of all titles and abstracts retrieved from electronic databases to determine their relevance. We excluded reports that were clearly irrelevant and retrieved the full texts of the remaining articles. These two review authors independently assessed the full-text manuscripts against the inclusion criteria. When necessary, a third reviewer (JSC) acted as an arbitrator to resolve disagreements that the two review authors could not resolve through discussion. At each stage, we detailed the number of studies selected in the PRISMA flowchart.

### Eligibility criteria

Inclusion criteria: The inclusion criteria are described using the following PICOS acronym. Participants: Patients diagnosed with MDD or bipolar depression (BD) according to the Diagnostic and Statistical Manual of Mental Disorders, 4th Edition, Text Revision (DSM-IV-TR), the Diagnostic and Statistical Manual of Mental Disorders, 5th Edition (DSM-5), or the International Classification of Diseases, 11th Revision (ICD-11). Intervention and Comparison: We compared MST with ECT treatment. Outcomes: The primary outcome measures were symptom severity, assessed using validated psychometric scales, including the Hamilton Rating Scale for Depression (HAMD/HDRS) and the Beck Depression Inventory (BDI). These variables were analyzed as continuous variables. Key secondary outcome measures were assessed using validated neuropsychological tests, including (but not limited to) the Montreal Cognitive Assessment (MoCA) and the Repeatable Battery for Assessment of Neuropsychological Status (RBANS). Study Design: Only randomized controlled trials evaluating the efficacy and safety of MST versus ECT were included. Exclusion criteria: (1) Lack of full text or raw data; (2) High risk of bias: Studies were assessed using the Cochrane Risk of Bias Assessment Tool; studies with four or more items rated as high risk were excluded; (3) Duplicate publications; (4) Animal studies, observational studies, reviews and meta-analyses, case reports, and non-randomized controlled trials; (5) Studies with significantly divergent outcome measures that cannot be pooled for effect size analysis (e.g., mismatched outcome measures, such as differing definitions of study outcomes).

### Data extraction

The two review authors (YHC, XZ) independently extracted the following data from the studies using data extraction forms: authors, year of publication, study design, sample size, mean age, duration of illness, clinical measures, ECT parameters, MST parameters, and duration of treatment. We extracted test scores accompanied by standard deviation (SD), sample size, and p-values to calculate effect sizes (ES). Outcome measures included total scores on depression scales and scores on various dimensions of neuropsychological assessments, such as immediate and delayed word recall, immediate and delayed visuospatial memory, and verbal fluency. When necessary, a third reviewer (JSC) served as an arbitrator to resolve disagreements that the two reviewers could not resolve through discussion. We included these details in the study characteristics tables (**Tables 1**, **2**).

**Table 1:** Characteristics of included studies.

Study (Country)	Diagnostic criteria	Diagnosis (100%)	Sample size		Sex ratio (male/female)		Average age (year)		Duration of illness or current depressive episode		Rating scale	Cognitive assessment scale
			Test group (MST)	Control group (ECT)	Test group (MST)	Control group (ECT)	Test group (MST)	Control group (ECT)	Test group (MST)	Control group (ECT)		
Wei Wang et al., 2026 (China)	DSM-5 BDI-II ≥ 29	TRD MDD (100)	60	60	12/48	13/47	15.20 ± 1.75	15.15 ± 1.64	14.52 ± 13.06 (months)	12.42 ± 13.28 (months)	BDI-II	MoCA
Wei Wang et al., 2025 (China)	ICD-11 HAMD ≥ 8	TRD MDD (100)	42	44	13/29	14/30	28.6 ± 13.8	30.7 ± 14.7	15. 9 ± 13. 2 (months)	18. 2 ± 13. 4 (months)	HAMD	MoCA
Zhi-De Deng et al., 2024 (USA)	DSM-IV-TR HDRS-24 ≥ 18	MDE MDD (86.3) and BD (13.7)	35	38	13/22	19/19	47.7 ± 15.6	48.2 ± 12.8	135.2 ± 208.3 (weeks)	114.5 ± 129.4 (weeks)	HDRS-24	–
Paul B. Fitzgerald et al., 2017 (Australia)	DSM-IV HAMD > 18	MDD (83.8) BD (16.2)	18	19	10/8	6/13	44.6 ± 14.8	47.2 ± 16.1	22.7 ± 14.3 (years)	27.6 ± 14.4 (years)	HAMD	–
Yang Qiao et al., 2025 (China)	DSM-5 HAMD17 ≥ 24	MDE MDD (42.5) and BD (57.5)	20	20	9/11	8/12	32.0 ± 18.5	35.7 ± 25.2	102.0 ± 115.6 (months)	52.3 ± 70.4 (months)	HAMD17	RBANS
Shawn M. McClintock et al., 2024 (USA)	DSM-IV-TR HDRS-24 ≥ 18	MDE MDD (86.3) and BD (13.7)	35	38	22/13	19/19	47.7 ± 15.6	48.2 ± 12.8	135.2 ± 208.3 (weeks)	114.5 ± 129.4 (weeks)	–	multiple neurocognitive domains

DSM‐IV‐TR, the Diagnostic and Statistical Manual of Mental Disorders, 4th Edition, Text Revision; DSM‐5, the Diagnostic and Statistical Manual of Mental Disorders, 5th Edition; ICD-11, the International Classification of Diseases, 11th Revision; HAMD/HDRS, the Hamilton Rating Scale for Depression; BDI, the Beck Depression Inventory.

**Table 2:** Specific parameters of ECT and MST.

Study (Country)	ECT parameters			MST parameters				Frequency
	Equipment	Stimulus setting	Stimulation pulse width	Equipment	Location	Stimulation intensity	Duration	
Wei Wang et al., 2026 (China)	Thymatron IV device	BLECT	–	MagSuper NS7000 device	International Standard 10–20 EEG system and vertex stimulation on Cz	100Hz 100%	8-10s	8-12
Wei Wang et al., 2025 (China)	Thymatron IV device	BLECT	–	MagSuper NS7000 device	International Standard 10–20 EEG system and vertex stimulation on Cz	100Hz 100%	8-10s	12-16
Zhi-De Deng et al., 2024 (USA)	spECTrum 5000Q	RULECT	0.3ms	Magstim Theta device (Magstim Co Ltd), double coil (44-mm inner diameter; 120-mm outer diameter)	The center of the coil is placed at the vertex	100Hz 100%	10s	-8
Paul B. Fitzgerald et al., 2017 (Australia)	Thymatron IV (Somatics, LLC, USA)	RULECT	1.0ms	Magventure A/S (Denmark), 130mm in diameter	the two coils fitted to the left and right of the vertex with the adjacent edges of the coils sitting side by side immediately above the vertex	100Hz 100%	commencing at 2s and increasing each time by 2s	-15
Yang Qiao et al., 2025 (China)	Thymatron IV (Somatics, LLC, USA)	–	1.0ms	Magpro X100 (MagVenture A/S)	–	100Hz 100%	up to 10s	8-12
Shawn M. McClintock et al., 2024 (USA)	spECTrum 5000Q or Thymatron IV (Somatics, Inc., LLC)	RULECT	0.3ms or 0.25ms	Magstim Theta device (Magstim Co Ltd), double coil (44-mm inner diameter; 120-mm outer diameter)	The center of the coil is placed at the vertex	100Hz 100%	5-10s	8

RULECT, right unilateral ECT; BLECT, bilateral ECT.

### Risk of bias assessment

The two review authors (YHC, XZ) assessed the quality of the included randomized controlled trials using the revised Cochrane Randomized Trials Risk of Bias Tool (ROB 2.0) (18). The ROB 2.0 tool assesses bias across the following domains: randomization process, deviation from the intended intervention, missing outcome data, outcome measurement, and outcome selection for reporting. The risk of bias in each domain is judged as low risk of bias, some concerns, or high risk of bias. Disagreements among reviewers were resolved through discussion, consensus, or arbitration by a third reviewer (JSC).

STATA 14.0 statistical software was used to generate funnel plots and bias scores to assess publication bias. When data in the funnel plot are roughly symmetrically distributed, there is no apparent publication bias; the opposite is true. Egger’s linear regression was used to test the symmetry of the funnel plot, and a p-value of <0.05 was considered indicative of significant asymmetry.

### Statistical analysis

Risk of bias in all eligible studies included in the review was assessed using Review Manager 5.3 software. The degree of heterogeneity among studies was evaluated by combining the I² statistic and P-value: I² ≥ 50% or P < 0.05 indicated high heterogeneity, and sensitivity analyses were conducted to identify the causes of heterogeneity; a random-effects model was used for the meta-analysis. I² < 50% or P > 0.1 indicated that the studies were homogeneous, and a fixed-effects model was used. The outcome measures included in this study were continuous variables. Since the scores on each test were continuous and the versions of the scales used varied across studies, the standardized mean difference (SMD) was selected as the pooled effect size. The primary outcomes were the change scores on the depression scale and the scores on each dimension of the neuropsychological assessment. This difference was statistically significant at P < 0.05.

## Results

### Study selection

**Figure 1:**
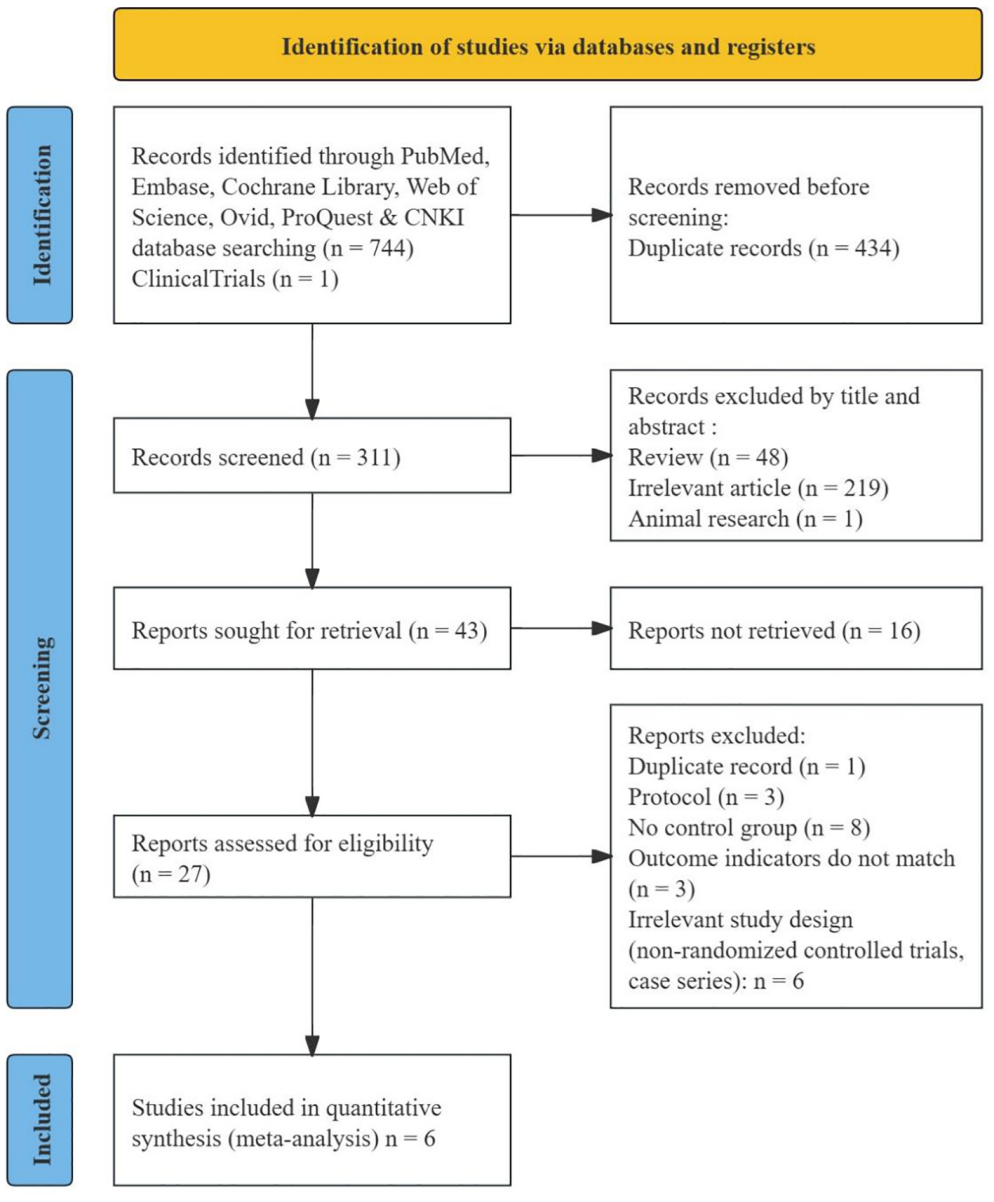
Flow diagram depicting the study selection process.

A total of 744 articles were included, sourced from PubMed, Embase, the Cochrane Library, Web of Science, Ovid, ProQuest, and the CNKI database. Additionally, one study was identified through ClinicalTrials. After removing duplicates, 311 articles were selected. After reviewing the titles and abstracts, a total of 268 ineligible studies were excluded, including 48 review and meta-analysis articles, 219 irrelevant studies, and 1 animal study, leaving 43 eligible studies. After excluding 16 articles that could not be retrieved, the full texts of 27 articles were reviewed. Among these, 1 was a duplicate, 3 were protocols, 8 lacked a control group, and 3 had outcome measures or study designs that did not meet the inclusion criteria. Ultimately, a meta-analysis was conducted on 6 randomized controlled trials involving 429 participants. The literature screening process is shown in **Figure 1**.

### Participants and study characteristics

The six randomized controlled trials ultimately included encompassed data from multiple countries, including China, the United States, and Australia, with publication dates ranging from 2017 to 2026. The sample sizes of the studies ranged from 38 to 120 participants, and most studies showed a higher proportion of female participants. The mean age of patients spanned a wide range, encompassing adolescents through middle-aged and older adults. One study focused on adolescents with depression, with mean ages of 15.15 ± 1.64 years in the ECT group and 15.20 ± 1.75 years in the MST group (19). Four studies reported the average duration of illness; among them, the MST group in Qiao’s study (20) had a duration as long as 102.0 ± 115.6 months. Regarding intervention protocols, the number of ECT or MST sessions did not exceed 16. MST stimulation parameters were generally 100 Hz, 100% output intensity, lasting 5–10 seconds; ECT employed right unilateral (RUL) or bilateral (BL) stimulation. The basic characteristics of the literature are shown in **Table 1**, and the specific parameters of ECT and MST stimulation are shown in **Table 2**.

### Quality assessment

**Figure 2:**
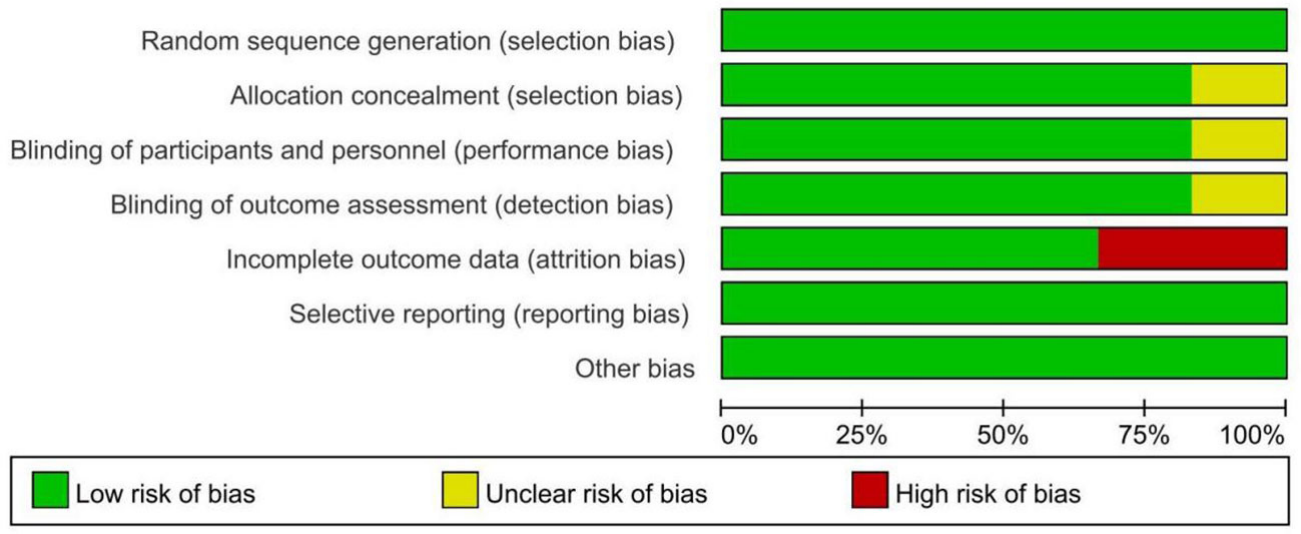
Risk of bias graph: review authors’ judgements about each risk of bias item presented as percentages across all included studies.

**Figure 3:**
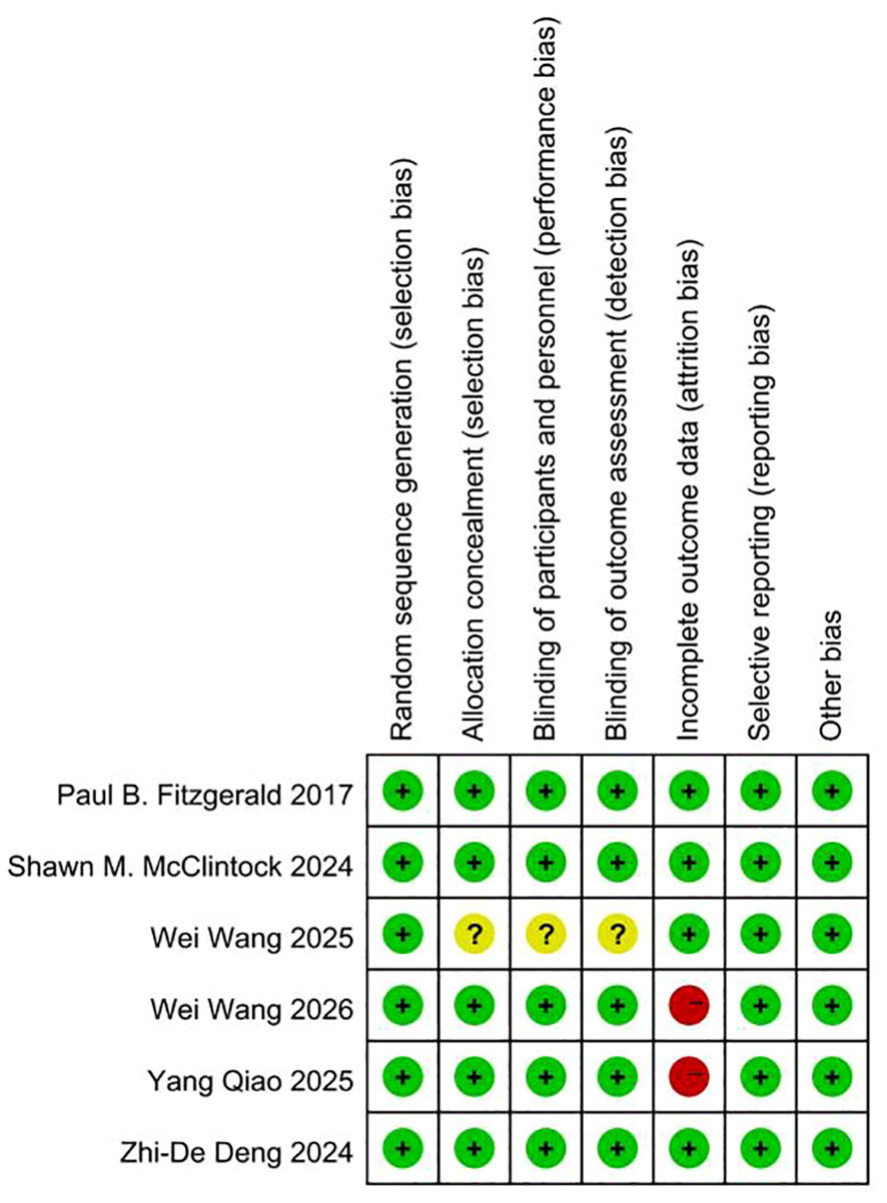
Risk of bias summary: review authors’ judgements about each risk of bias item for each included study.

Each bias risk item (**Figure 2**) and each study (**Figure 3**) provides a graphical representation of the overall risk of bias in the included studies. Among the six studies ultimately included, two reported missing subjects but had relatively complete test results (19, 20); one study mentioned randomization but did not describe the allocation method, and was assessed as “unknown risk”; furthermore, this study did not mention blinding of participants, and was assessed as “unknown risk” (21). The remaining studies demonstrated higher quality.

### Antidepressant outcomes

**Figure 4:**
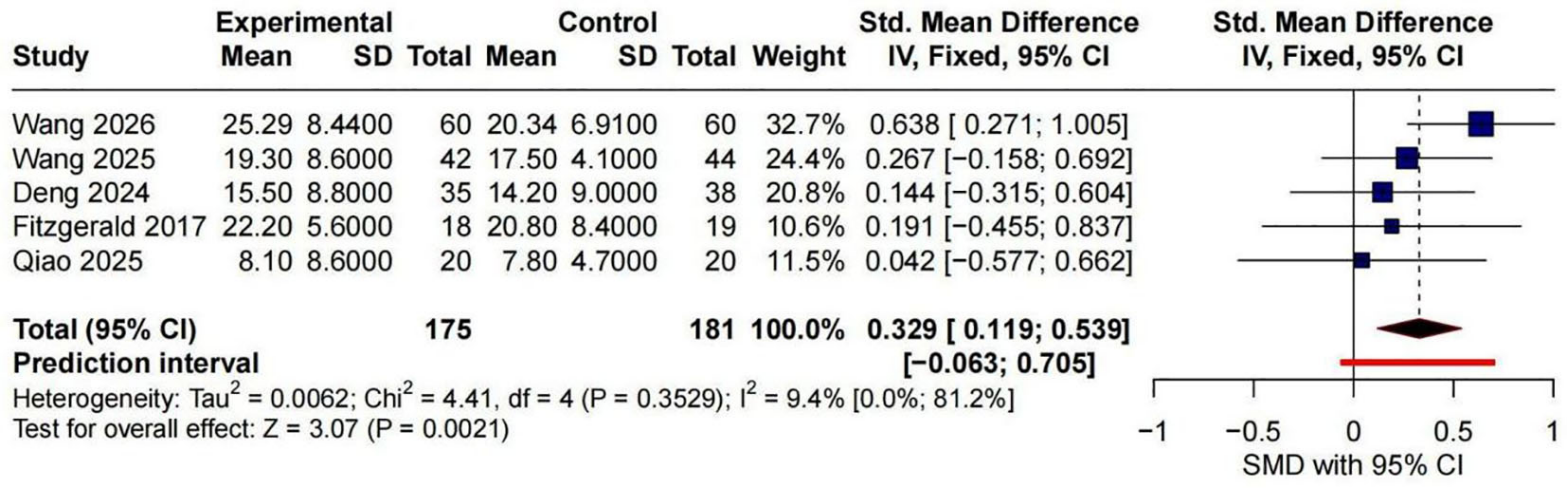
Forest plot of the efficacy of MST and ECT in the treatment of depression. Experimental refers to MST, while Control refers to ECT.

Results of a meta-analysis on the efficacy of MST for treating depression. Four studies used the HAMD/HDRS scales, and one study used the BDI-II scale to assess changes in scores as a measure of treatment efficacy for depressive symptoms; a total of 356 patients were included (19–23). As shown in **Figure 4**, the difference in scale score changes between MST and ECT was statistically significant; post-treatment scale scores in the MST group were 0.329 higher than those in the ECT group (95% CI: 0.119–0.539, I² = 9.4%, P = 0.0021), suggesting that the antidepressant efficacy of MST was significantly inferior to that of ECT.

### Cognitive performance

**Figure 5:**
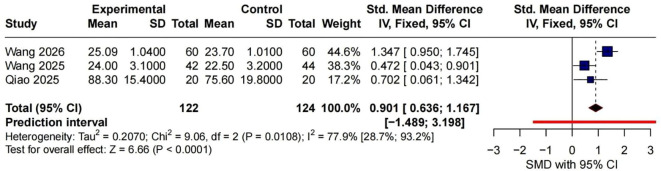
Forest plot of the scale assessing the effect of MST antidepressant treatment on cognitive performance.

Three studies used different scales to assess overall cognitive function following MST and ECT treatment, involving a total of 246 patients (19–21). The results consistently showed that the MST group had higher cognitive function scores than the ECT group; however, there was a high degree of heterogeneity among the studies (**Figure 5**).

**Figure 6:**
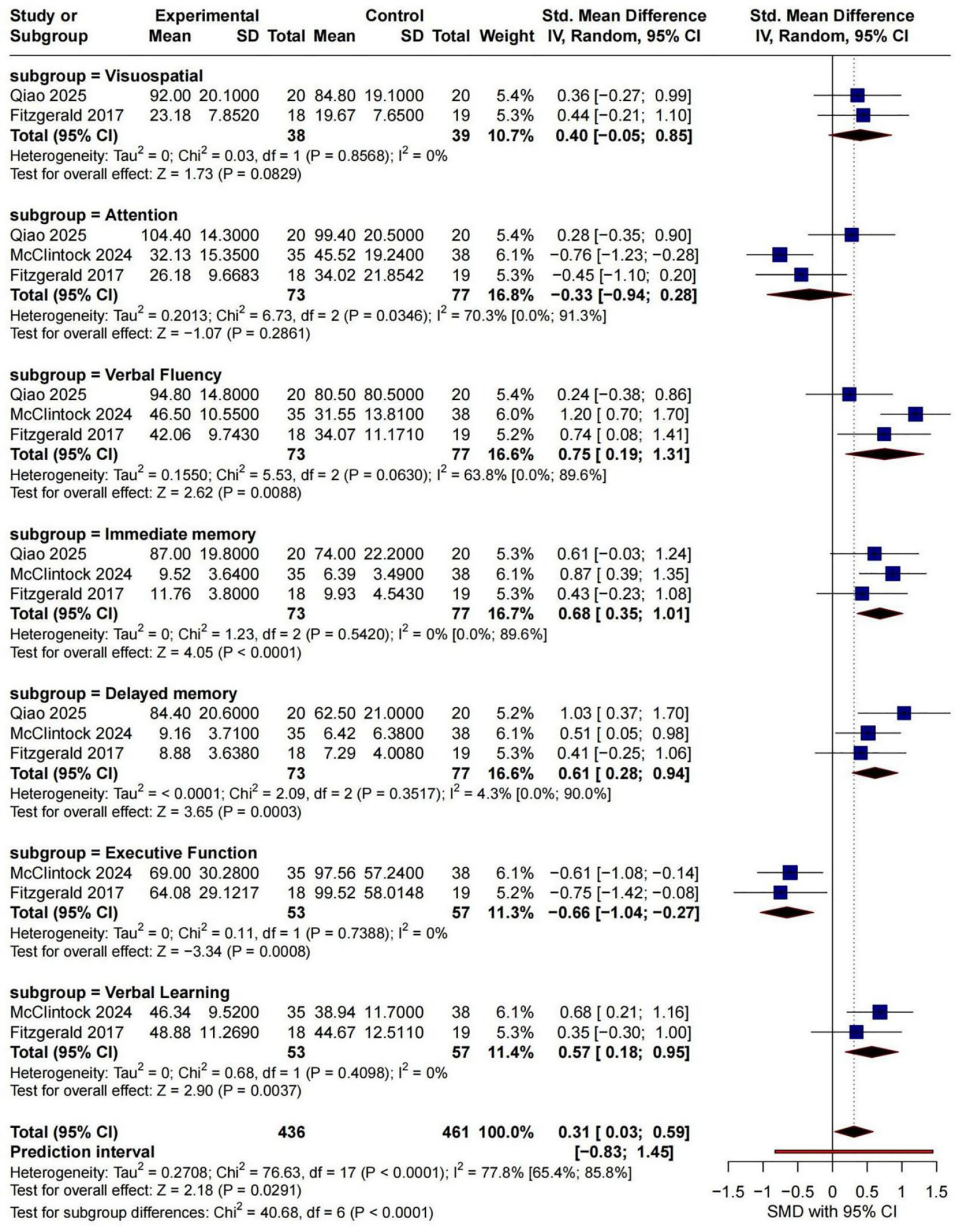
Forest plot of the impact of MST antidepressant treatment on neurocognitive assessment scores. Experimental refers to MST, while Control refers to ECT.

In the subgroup analysis of neurocognitive assessments (**Figure 6**), three studies (n = 150) reported on Attention and Visuospatial function (20, 22, 24), and the results showed no statistically significant differences between the MST and ECT groups. Regarding verbal fluency, the MST group scored significantly higher than the ECT group by 0.75 (95% CI: 0.19–1.31, Z = 2.62, P = 0.0088), although heterogeneity was present among the studies. For immediate memory and delayed memory, three studies (n=150) (20, 22, 24) showed that the MST group scored higher than the ECT group by 0.68 (95% CI: 0.35–1.01, Z = 4.05, P<0.0001) and 0.61 (95% CI: 0.28–0.94, Z = 3.65, P = 0.0003), respectively. Regarding executive function, two studies (n=110) (22, 24) indicated that the MST group scored 0.66 points lower than the ECT group (95% CI: -1.04 to -0.27, Z=-3.34, P = 0.0008); whereas for Verbal Learning, the MST group scored 0.57 points higher than the ECT group (95% CI: 0.18–0.95, Z = 2.90, P = 0.0037).

### Publication bias

**Figure 7:**
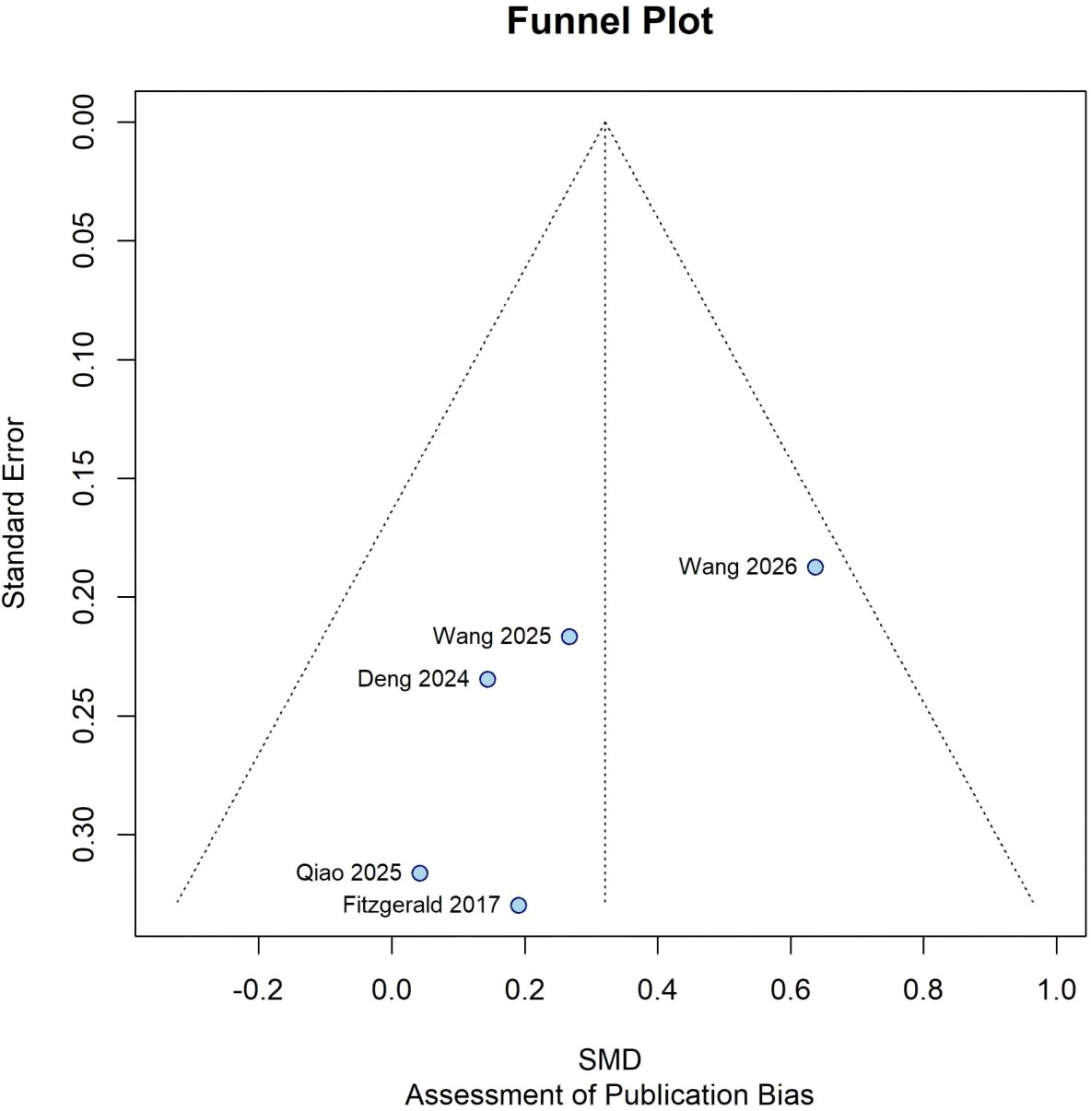
Funnel plot of depression scale scores.

A funnel plot of depression scale scores (**Figure 7**) was used to conduct a qualitative assessment of publication bias in the included studies; the results showed an asymmetrical distribution of data points. A quantitative assessment was further conducted using Egger’s regression test, which revealed no significant publication bias (P = 0.139).

## Discussion

This latest systematic review and meta-analysis included six randomized controlled trials involving 429 patients with major depressive disorder (MDD) and compared the clinical efficacy of MST versus ECT for the treatment of depression, as well as their effects on cognitive function. The results of this study indicate that, in terms of antidepressant efficacy, MST significantly reduced depression scale scores, suggesting it has antidepressant effects; however, compared with ECT, the antidepressant efficacy of MST was lower, and the difference was statistically significant. Cognitive function assessments revealed that the MST group scored significantly higher than the ECT group on multiple tests, including Verbal Fluency, Immediate Memory, Delayed Memory, and Verbal Learning, suggesting that MST causes relatively less cognitive impairment.

Our analysis suggests that the key reason for the discrepancy between the findings of this study and previous understanding lies in the fact that this meta-analysis included, for the first time, a large-sample randomized controlled trial conducted in China targeting adolescents with MDD (19). This study has two notable characteristics: the study population consisted of adolescents, and its sample size was the largest among those included in the current analysis. The results of this study indicated that the ECT group showed a slightly greater reduction in post-treatment depression scale scores than the MST group, but the difference in clinical remission rates between the two groups was not significant. Given its high weighting in the pooled analysis, this effect was amplified, thereby largely dictating the final results of this meta-analysis and shifting the overall effect size in favor of ECT. In contrast, the previous meta-analysis (15), which lacked such large-sample adolescent data, reflected the characteristics of studies primarily involving adult populations; these studies tended to report no statistically significant difference in efficacy between the two treatment modalities.

Although we hypothesized that the antidepressant efficacy of MST and ECT might vary across different age groups, the reality is more complex. A recent prospective study (25) suggests that MST demonstrates good efficacy and tolerability in the treatment of major depressive disorder across all age groups, with particular advantages observed in adolescents. It is speculated that these age-related differences in efficacy may stem from distinct neurobiological characteristics, namely that the prefrontal cortex in adolescents is still in a developmental stage (26). Following MST, microstructural changes were observed in multiple white matter tracts (the right single tract and the left superior longitudinal tract), primarily manifested as changes in diffusion measures such as fractional anisotropy (FA). The increase in FA in the right tract following aMST may reflect enhanced microstructural organization in emotional regulation pathways, thereby serving as a response to symptom improvement in adolescents (27). However, another recent observational study indicated that standardized ECT protocols are more effective in adults than in adolescents (28). It is speculated that adolescents’ poorer treatment response may be closely related to their developmental stage and physiological characteristics. The adolescent brain is still undergoing functional and structural development, particularly as connections between the prefrontal cortex and the limbic system remain immature (29). Under emotional stress, adolescents often lack effective coping strategies (30), which may weaken their positive response to ECT. Additionally, they are more prone to experiencing anxiety and fear during treatment, thereby reducing overall efficacy.

Consequently, due to neurobiological differences, current evidence has not yet reached a consistent conclusion regarding the differences in antidepressant efficacy between MST and ECT across different age groups. Current research lacks randomized controlled trials directly comparing antidepressant efficacy differences across different age groups. Future studies should incorporate more rigorously designed randomized controlled trials with pre-specified age-stratified analyses to clarify whether age serves as a factor influencing antidepressant efficacy, thereby providing a more robust evidence base for individualized clinical treatment decisions.

In this meta-analysis, three randomized controlled trials reported the positive effects of MST and ECT on cognitive function using cognitive assessment scales; however, there was a high degree of heterogeneity among the studies. Further subgroup analysis revealed that MST demonstrated significantly better outcomes than ECT in certain cognitive domains, particularly memory and verbal function, which is consistent with the findings of previous studies. The differences in cognitive effects between MST and ECT may stem from their distinct mechanisms of action on brain function. In terms of stimulation depth, MST utilizes a magnetic field with lower attenuation, enabling it to more precisely induce epileptic activity in the superficial layers of the cortex, which is generally considered a key factor in its cognitive protective effects (31–33), as the magnetic field can penetrate only about 2 centimeters below the cortical surface (34, 35), thereby potentially avoiding stimulation of subcortical medial-temporal structures (such as the hippocampus) (36). In terms of stimulation breadth, computer modeling studies indicate that MST primarily stimulates the cerebral cortex and approximately 21% of the brain’s volume (37, 38), ECT delivers stimulation via two electrodes to achieve therapeutic effects, resulting in a broader range of influence that involves deep brain regions such as the hippocampus; this may be the reason for the memory impairment it causes (39). Clinical evidence suggests that even with RUL electrode placement, the electric field induced by ECT remains relatively broad, with a penetration range extending across the contralateral hemisphere and subcortical structures (40, 41). Recent studies have found that the sequential activation of nodes within the language network exhibits high temporal precision, with alpha and beta rhythmic activity playing a key role in the direction of network propagation (42). Changes in alpha and beta rhythmic activity induced by ECT may explain the deterioration of patients’ verbal function (43). Furthermore, patients’ reasoning and problem-solving abilities improved following ECT treatment (44). This is not surprising, as previous studies have also reported improvements in these cognitive domains (45). Therefore, reasoning and problem-solving abilities may be more stable compared to other cognitive domains, and the impact of ECT on these abilities is limited. However, overall, the number of studies included in the subgroup analyses of this meta-analysis was small, highlighting the need for more large-scale randomized clinical trials to definitively assess the cognitive differences between ECT and MST. Future research should further explore the specific effects of MST on different cognitive dimensions, not limited to memory, but rather evaluating its impact on cognitive function from a more comprehensive perspective.

This study has the following limitations. First, due to the limited number of published studies comparing MST and ECT, the sample size included in the analysis was relatively small, which may affect statistical power and the stability of effect estimates. Second, among the included original studies, only one focused on adolescents, limiting this study’s ability to conduct subgroup analyses of age-related differences in efficacy. Furthermore, this study used the BDI-II scale for the diagnosis of MDD. As a self-report scale, it relies on individuals’ self-reports and may yield a certain proportion of false-positive results. Third, the included studies evaluated only short-term cognitive function following treatment and lacked long-term follow-up data; therefore, the long-term effects of the two treatment modalities on cognitive function require further investigation. Finally, the cognitive assessment tools used in the original studies varied, leading to some heterogeneity in the pooled results for certain cognitive outcome measures.

## Conclusions

Our analysis suggests that MST appears to have similar antidepressant effects to ECT for depression, although its efficacy is somewhat less than that of ECT. Furthermore, MST causes less cognitive impairment than ECT. There is currently no definitive evidence to support MST as the preferred physical therapy for depression in place of ECT. Future large-scale randomized controlled trials targeting different age groups, employing high-quality study designs and long-term follow-up, are needed to further validate its clinical value and clinical role.

## Data Availability

The original contributions presented in the study are included in the article/**Supplementary Material**, further inquiries can be directed to the corresponding author/s.
