# Supplementary material for: Comparative efficacy and cognitive function of magnetic seizure therapy and electroconvulsive therapy for major depressive disorder: a systematic review and meta-analysis of randomized controlled trials
Source: Front Psychiatry. 2026 Jul 22;17:1886955. doi: 10.3389/fpsyt.2026.1886955 (PMC13440948; doi:10.3389/fpsyt.2026.1886955)
Supplement: Supplementary file 1 [file Table1.docx]

Supplementary Material

PubMed

((("Depressive Disorder, Major"[Mesh]) OR ((((((((Major Depressive Disorders[Title/Abstract]) OR (Clinical Depression[Title/Abstract])) OR (Major Depressive Disorder[Title/Abstract])) OR (MDD[Title/Abstract])) OR (Depressive Disorders[Title/Abstract])) OR (Disorder, Depressive[Title/Abstract])) OR (Disorders, Depressive[Title/Abstract])) OR (Depression[Title/Abstract]))) AND ((magnetic seizure therapy[Title/Abstract]) OR (MST[Title/Abstract]))) AND (hiru.mcmaster.ca/-Therapy randomized controlled trial[Publication Type] OR randomized[Title/Abstract] OR placebo[Title/Abstract])

Embase

#1 'depressive disorders, major':ab,ti OR 'major depressive disorders':ab,ti OR 'clinical depression':ab,ti OR 'major depressive disorder':ab,ti OR 'mdd':ab,ti OR 'depressive disorders':ab,ti OR 'disorder, depressive':ab,ti OR 'disorders, depressive':ab,ti OR 'depression':ab,ti

#2 'magnetic seizure therapy':ab,ti OR 'mst':ab,ti

#3 'randomized controlled trial':ab,ti OR 'randomized':ab,ti OR 'placebo':ab,ti OR 'rct':ab,ti

#4	#1 AND #2 AND #3

Web of Science

#1 TS=(Depressive Disorders, Major OR Major Depressive Disorders OR Clinical Depression OR Major Depressive Disorder OR MDD OR Depressive Disorders OR Disorder, Depressive OR Disorders, Depressive OR Depression)

#2 TS=(magnetic seizure therapy OR MST)

#3 TS=(randomized controlled trial OR randomized OR placebo OR RCT)

#4 #1 AND #2 AND #3

Cochrane Library

#1 (Depressive Disorders, Major):ab,ti,kw OR (Major Depressive Disorders):ab,ti,kw OR (Clinical Depression):ab,ti,kw OR (Major Depressive Disorder):ab,ti,kw OR (MDD):ab,ti,kw OR (Depressive Disorders):ab,ti,kw OR (Disorder, Depressive):ab,ti,kw OR (Disorders, Depressive):ab,ti,kw OR (Depression):ab,ti,kw

#2 (magnetic seizure therapy):ab,ti,kw OR (MST):ab,ti,kw

#3 (randomized controlled trial):ab,ti,kw OR (randomized):ab,ti,kw OR (placebo):ab,ti,kw OR (RCT):ab,ti,kw

#4 #1 AND #2 AND #3

Ovid

#1 Depressive Disorders, Major OR Major Depressive Disorders OR Clinical Depression OR Major Depressive Disorder OR MDD OR Depressive Disorders OR Disorder, Depressive OR Disorders, Depressive OR Depression

#2 magnetic seizure therapy OR MST

#3 randomized controlled trial OR randomized OR placebo OR RCT

#4 #1 AND #2 AND #3

ProQuest

#1 Depressive Disorders, Major OR Major Depressive Disorders OR Clinical Depression OR Major Depressive Disorder OR MDD OR Depressive Disorders OR Disorder, Depressive OR Disorders, Depressive OR Depression

#2 magnetic seizure therapy OR MST

#3 randomized controlled trial OR randomized OR placebo OR RCT

#4 #1 AND #2 AND #3

CNKI

#1 Depressive Disorders, Major OR Major Depressive Disorders OR Clinical Depression OR Major Depressive Disorder OR MDD OR Depressive Disorders OR Disorder, Depressive OR Disorders, Depressive OR Depression

#2 magnetic seizure therapy OR MST

#3 randomized controlled trial OR randomized OR placebo OR RCT

#4 #1 AND #2 AND #3
